# Supplementary material for: Immersive Virtual Reality as Analgesia during Dressing Changes of Hospitalized Children and Adolescents with Burns: A Systematic Review with Meta-Analysis
Source: Children (Basel). 2020 Oct 22;7(11):194. doi: 10.3390/children7110194 (PMC7690261; doi:10.3390/children7110194)
Supplement: Supplementary file 1 [file children-07-00194-s001.pdf]

# **Table S1.** search strategy PubMed

| Search | Query | Items found | Time |
|---|---|---|---|
| | **Search 22-03-2020 (after re-run)** | | |
| **Search** | **Query** | **Items found** | **Time** |
| #99 | Search (((((((((((((((((((((child[MeSH Terms]) OR child) OR children) OR child, preschool[MeSH Terms]) OR preschool child) OR preschool children) OR pediatrics[MeSH Terms]) OR pediatric) OR adolescent[MeSH Terms]) OR adolescents) OR teens) OR teen) OR teenagers) OR teenager) OR minors[MeSH Terms]) OR minor)) AND (((((((((((((burns[MeSH Terms]) OR burns, chemical[MeSH Terms]) OR burns, electric[MeSH Terms]) OR burn) OR burns) OR burn injury) OR burn injuries) OR burn, electric) OR burn, chemical) OR scald) OR scalds) OR debridement[MeSH Terms]) OR debridements)) AND (((((((((((hospitalization[MeSH Terms]) OR hospitalizations) OR child, hospitalized[MeSH Terms]) OR children, hospitalized) OR hospitals[MeSH Terms]) OR hospital) OR inpatients[MeSH Terms]) OR inpatient) OR burn units[MeSH Terms]) OR burn unit) OR burn center))) AND ((((((((((((((((((((virtual reality[MeSH Terms]) OR virtual reality) OR virtual realities) OR virtual reality therapy) OR virtual reality therapies) OR virtual reality immersion therapy) OR computer simulation) OR computer simulations) OR multimedia) OR multimedium) OR application, mobile) OR applications, mobile) OR mobile apps) OR portable software apps) OR computer assisted therapy) OR computer assisted therapies) OR virtual reality exposure therapy[MeSH Terms]) OR multimedia[MeSH Terms]) OR mobile applications[MeSH Terms]) OR therapy, computer-assisted[MeSH Terms])) AND ((((((((((((((((((((((((((((((((pain[MeSH Terms]) OR pain) OR pains) OR acute pain[MeSH Terms]) OR acute pain) OR pain, procedural[MeSH Terms]) OR pain, procedural) OR breakthrough pain) OR anxiety[MeSH Terms]) OR anxiety) OR fear[MeSH Terms]) OR fear) OR fears) OR painful[MeSH Terms]) OR painful) OR distress) OR pain perception[MeSH Terms]) OR pain perception) OR pain perceptions) OR pain management[MeSH Terms]) OR pain management) OR painful procedures[MeSH Terms]) OR painful procedures) OR severe pain) OR pain distraction children[MeSH Terms]) OR pain distraction children) OR self-reported pain[MeSH Terms]) OR self-reported pain) OR observer-reported pain[MeSH Terms]) OR observer-reported pain) OR behavioral measures of pain[MeSH Terms]) OR behavioral measures of pain) | 41 | 8:31:53 |
| #98 | Search ((((((((((((((((((child[MeSH Terms]) OR child) OR children) OR child, preschool[MeSH Terms]) OR preschool child) OR preschool children) OR pediatrics[MeSH Terms]) OR pediatric) OR adolescent[MeSH Terms]) OR adolescents) OR teens) OR teen) OR teenagers) OR teenager) OR minors[MeSH Terms]) OR minor)) AND (((((((((((((burns[MeSH Terms]) OR burns, chemical[MeSH Terms]) OR burns, electric[MeSH Terms]) OR burn) OR burns) OR burn injury) OR burn injuries) OR burn, electric) OR burn, chemical) OR scald) OR scalds) OR debridement[MeSH Terms]) OR debridements)) AND (((((((((((hospitalization[MeSH Terms]) OR hospitalizations) OR child, hospitalized[MeSH Terms]) OR children, hospitalized) OR hospitals[MeSH Terms]) OR hospital) OR inpatients[MeSH Terms]) OR inpatient) OR burn units[MeSH Terms]) OR burn unit) OR burn center) | 16552 | 8:31:34 |

| | | | |
|---|---|---|---|
| #97 | Search (((((((((((((((((((((((((((((((pain[MeSH Terms]) OR pain) OR pains) OR acute pain[MeSH Terms]) OR acute pain) OR pain, procedural[MeSH Terms]) OR pain, procedural) OR breakthrough pain) OR anxiety[MeSH Terms]) OR anxiety) OR fear[MeSH Terms]) OR fear) OR fears) OR painful[MeSH Terms]) OR painful) OR distress) OR pain perception[MeSH Terms]) OR pain perception) OR pain perceptions) OR pain management[MeSH Terms]) OR pain management) OR painful procedures[MeSH Terms]) OR painful procedures) OR severe pain) OR pain distraction children[MeSH Terms]) OR pain distraction children) OR self-reported pain[MeSH Terms]) OR self-reported pain) OR observer-reported pain[MeSH Terms]) OR observer-reported pain) OR behavioral measures of pain[MeSH Terms]) OR behavioral measures of pain | 1207617 | 8:31:01 |
| #96 | Search behavioral measures of pain | 11419 | 8:29:12 |
| #95 | Search behavioral measures of pain[MeSH Terms] | 6413 | 8:29:06 |
| #94 | Search observer-reported pain | 30 | 8:28:47 |
| #93 | Search observer-reported pain[MeSH Terms] | 16 | 8:28:39 |
| #92 | Search self-reported pain | 22957 | 8:28:24 |
| #91 | Search self-reported pain[MeSH Terms] | 10904 | 8:28:18 |
| #90 | Search pain distraction children | 594 | 8:27:25 |
| #89 | Search pain distraction children[MeSH Terms] | 466 | 8:27:20 |
| #88 | Search severe pain | 70684 | 8:26:39 |
| #87 | Search painful procedures | 381130 | 8:26:30 |
| #86 | Search painful procedures[MeSH Terms] | 39448 | 8:26:26 |
| #85 | Search pain management | 126092 | 8:26:00 |
| #84 | Search pain management[MeSH Terms] | 32754 | 8:25:53 |
| #83 | Search pain perceptions | 23905 | 8:25:33 |
| #82 | Search pain perception | 20396 | 8:25:25 |
| #81 | Search pain perception[MeSH Terms] | 4858 | 8:25:14 |
| #80 | Search distress | 125709 | 8:24:58 |
| #79 | Search painful | 840995 | 8:24:50 |
| #78 | Search painful[MeSH Terms] | 388609 | 8:24:44 |
| #77 | Search fears | 82825 | 8:24:26 |
| #76 | Search fear | 75534 | 8:24:21 |
| #75 | Search fear[MeSH Terms] | 32227 | 8:24:16 |
| #74 | Search anxiety | 233847 | 8:24:05 |
| #73 | Search anxiety[MeSH Terms] | 82587 | 8:23:57 |
| #72 | Search breakthrough pain | 1499 | 8:23:46 |
| #71 | Search pain, procedural | 4085 | 8:23:27 |
| #70 | Search pain, procedural[MeSH Terms] | 348 | 8:23:16 |
| #69 | Search acute pain | 83405 | 8:22:45 |

| | | | |
|---|---|---|---|
| #68 | Search acute pain[MeSH Terms] | 2030 | 8:22:28 |
| #67 | Search pains | 819418 | 8:22:14 |
| #66 | Search pain | 815429 | 8:22:10 |
| #65 | Search pain[MeSH Terms] | 388609 | 8:22:04 |
| #64 | Search ((((((((((((((((((virtual reality[MeSH Terms]) OR virtual reality) OR virtual realities) OR virtual reality therapy) OR virtual reality therapies) OR virtual reality immersion therapy) OR computer simulation) OR computer simulations) OR multimedia) OR multimedium) OR application, mobile) OR applications, mobile) OR mobile apps) OR portable software apps) OR computer assisted therapy) OR computer assisted therapies) OR virtual reality exposure therapy[MeSH Terms]) OR multimedia[MeSH Terms]) OR mobile applications[MeSH Terms]) OR therapy, computer-assisted[MeSH Terms] | 374107 | 8:21:05 |
| #63 | Search therapy, computer-assisted[MeSH Terms] | 62399 | 8:20:01 |
| #62 | Search mobile applications[MeSH Terms] | 5338 | 8:19:31 |
| #61 | Search multimedia[MeSH Terms] | 1905 | 8:18:47 |
| #60 | Search virtual reality exposure therapy[MeSH Terms] | 581 | 8:18:33 |
| #59 | Search computer assisted therapies | 83652 | 8:17:47 |
| #58 | Search computer assisted therapy | 83286 | 8:17:32 |
| #57 | Search portable software apps | 10665 | 8:17:11 |
| #56 | Search mobile apps | 11852 | 8:16:48 |
| #55 | Search applications, mobile | 10655 | 8:16:41 |
| #54 | Search application, mobile | 18458 | 8:16:27 |
| #53 | Search multimedium | 6549 | 8:16:01 |
| #52 | Search multimedia | 6545 | 8:15:49 |
| #51 | Search computer simulations | 268212 | 8:15:41 |
| #50 | Search computer simulation | 254410 | 8:15:30 |
| #49 | Search virtual reality immersion therapy | 1001 | 8:15:20 |
| #48 | Search virtual reality therapies | 1094 | 8:15:01 |
| #47 | Search virtual reality therapy | 2666 | 8:14:49 |
| #46 | Search virtual realities | 83 | 8:14:33 |
| #45 | Search virtual reality | 11080 | 8:14:21 |
| #44 | Search virtual reality[MeSH Terms] | 1559 | 8:14:07 |
| #43 | Search (((((((((hospitalization[MeSH Terms]) OR hospitalizations) OR child, hospitalized[MeSH Terms]) OR children, hospitalized) OR hospitals[MeSH Terms]) OR hospital) OR inpatients[MeSH Terms]) OR inpatient) OR burn units[MeSH Terms]) OR burn unit) OR burn center | 5031415 | 8:12:51 |
| #42 | Search burn center | 13583 | 8:12:16 |
| #41 | Search burn unit | 7889 | 8:11:59 |
| #40 | Search burn units[MeSH Terms] | 2520 | 8:11:53 |

| | | | |
|---|---|---|---|
| #39 | Search inpatient | 114259 | 8:11:38 |
| #38 | Search inpatients[MeSH Terms] | 21091 | 8:11:33 |
| #37 | Search hospital | 4879551 | 8:11:23 |
| #36 | Search hospitals[MeSH Terms] | 270189 | 8:11:14 |
| #35 | Search children, hospitalized | 26811 | 8:10:55 |
| #34 | Search child, hospitalized[MeSH Terms] | 6572 | 8:10:39 |
| #33 | Search hospitalizations | 323395 | 8:10:21 |
| #32 | Search hospitalization[MeSH Terms] | 233131 | 8:10:02 |
| #31 | Search (((((((((((burns[MeSH Terms]) OR burns, chemical[MeSH Terms]) OR burns, electric[MeSH Terms]) OR burn) OR burns) OR burn injury) OR burn injuries) OR burn, electric) OR burn, chemical) OR scald) OR scalds) OR debridement[MeSH Terms]) OR debridements | 143952 | 8:09:30 |
| #30 | Search debridements | 32418 | 8:08:43 |
| #29 | Search debridement[MeSH Terms] | 16002 | 8:08:32 |
| #28 | Search scalds | 838 | 8:08:17 |
| #27 | Search scald | 1960 | 8:08:12 |
| #26 | Search burn, chemical | 9753 | 8:08:07 |
| #25 | Search burn, electric | 3496 | 8:07:54 |
| #24 | Search burn injuries | 100384 | 8:07:44 |
| #23 | Search burn injury | 100942 | 8:07:32 |
| #22 | Search burns | 98515 | 8:07:22 |
| #21 | Search burn | 113408 | 8:07:17 |
| #20 | Search burns, electric[MeSH Terms] | 2360 | 8:07:10 |
| #19 | Search burns, chemical[MeSH Terms] | 6370 | 8:06:54 |
| #18 | Search burns[MeSH Terms] | 57039 | 8:06:31 |
| #17 | Search (((((((((((((((child[MeSH Terms]) OR child) OR children) OR child, preschool[MeSH Terms]) OR preschool child) OR preschool children) OR pediatrics[MeSH Terms]) OR pediatric) OR adolescent[MeSH Terms]) OR adolescents) OR teens) OR teen) OR teenagers) OR teenager) OR minors[MeSH Terms]) OR minor | 3997042 | 8:05:58 |
| #16 | Search minor | 235099 | 8:05:02 |
| #15 | Search minors[MeSH Terms] | 2560 | 8:04:57 |
| #14 | Search teenager | 2048914 | 8:04:44 |
| #13 | Search teenagers | 2050371 | 8:04:36 |
| #12 | Search teen | 2049231 | 8:04:28 |
| #11 | Search teens | 2049561 | 8:04:24 |
| #10 | Search adolescents | 2078162 | 8:04:10 |
| #9 | Search adolescent[MeSH Terms] | 1993023 | 8:03:59 |
| #8 | Search pediatric | 780386 | 8:03:40 |

| | | | |
|---|---|---|---|
| #7 | Search pediatrics[MeSH Terms] | 57033 | 8:03:31 |
| #6 | Search preschool children | 908605 | 8:03:11 |
| #5 | Search preschool child | 906709 | 8:02:39 |
| #4 | Search child, preschool[MeSH Terms] | 903532 | 8:02:26 |
| #3 | Search children | 2452667 | 8:02:04 |
| #2 | Search child | 2200467 | 8:01:52 |
| #1 | Search child[MeSH Terms] | 1879831 | 8:01:44 |

**Table S2.** search strategy CINAHL

| Search 22-03-2020 (after re-run) | | | | |
|---|---|---|---|---|
| **Search** | **Query** | **Limiters/Expanders** | **Last Run Via** | **Results** |
| S16 | S6 AND S11 AND S12 AND S15 | Expanders - Apply equivalent subjects<br>Search modes - Boolean/Phrase | Interface - EBSCOhost Research Databases<br>Search Screen - Advanced Search<br>Database - CINAHL | 6 |
| S15 | S13 OR S14 | Expanders - Apply equivalent subjects<br>Search modes - Boolean/Phrase | Interface - EBSCOhost Research Databases<br>Search Screen - Advanced Search<br>Database - CINAHL | 215874 |
| S14 | MM Pain Management | Expanders - Apply equivalent subjects<br>Search modes - Boolean/Phrase | Interface - EBSCOhost Research Databases<br>Search Screen - Advanced Search<br>Database - CINAHL | 3611 |
| S13 | MH Pain+ | Expanders - Apply equivalent subjects<br>Search modes - Boolean/Phrase | Interface - EBSCOhost Research Databases<br>Search Screen - Advanced Search<br>Database - CINAHL | 214247 |

| | | | | |
|---|---|---|---|---|
| S12 | MH Virtual Reality+ | Expanders - Apply equivalent subjects<br>Search modes - Boolean/Phrase | Interface - EBSCOhost Research Databases<br>Search Screen - Advanced Search<br>Database - CINAHL | 5207 |
| S11 | S7 OR S8 OR S9 OR S10 | Expanders - Apply equivalent subjects<br>Search modes - Boolean/Phrase | Interface - EBSCOhost Research Databases<br>Search Screen - Advanced Search<br>Database - CINAHL | 14186 |
| S10 | MM Burn Patients | Expanders - Apply equivalent subjects<br>Search modes - Boolean/Phrase | Interface - EBSCOhost Research Databases<br>Search Screen - Advanced Search<br>Database - CINAHL | 675 |
| S9 | MM Burns, Chemical | Expanders - Apply equivalent subjects<br>Search modes - Boolean/Phrase | Interface - EBSCOhost Research Databases<br>Search Screen - Advanced Search<br>Database - CINAHL | 742 |
| S8 | MM Burns, Electric | Expanders - Apply equivalent subjects<br>Search modes - Boolean/Phrase | Interface - EBSCOhost Research Databases<br>Search Screen - Advanced Search<br>Database - CINAHL | 445 |

| | | | | |
|---|---|---|---|---|
| S7 | MM Burns | Expanders - Apply equivalent subjects<br>Search modes - Boolean/Phrase | Interface - EBSCOhost Research Databases<br>Search Screen - Advanced Search<br>Database - CINAHL | 12591 |
| S6 | S1 OR S2 OR S3 OR S4 OR S5 | Expanders - Apply equivalent subjects<br>Search modes - Boolean/Phrase | Interface - EBSCOhost Research Databases<br>Search Screen - Advanced Search<br>Database - CINAHL | 558765 |
| S5 | MH Adolescence+ | Expanders - Apply equivalent subjectsSearch modes - Boolean/Phrase | Interface - EBSCOhost Research DatabasesSearch Screen - Advanced SearchDatabase - CINAHL | 555459 |
| S4 | (MM "Minors (Legal)") | Expanders - Apply equivalent subjects<br>Search modes - Boolean/Phrase | Interface - EBSCOhost Research Databases<br>Search Screen - Advanced Search<br>Database - CINAHL | 407 |
| S3 | MM Child, Preschool | Expanders - Apply equivalent subjects<br>Search modes - Boolean/Phrase | Interface - EBSCOhost Research Databases<br>Search Screen - Advanced Search<br>Database - CINAHL | 374 |

| | | | | |
|---|---|---|---|---|
| S2 | MM Child, Hospitalized | Expanders - Apply equivalent subjects<br>Search modes - Boolean/Phrase | Interface - EBSCOhost Research Databases<br>Search Screen - Advanced Search<br>Database - CINAHL | 3338 |
| S1 | MM Child | Expanders - Apply equivalent subjects<br>Search modes - Boolean/Phrase | Interface - EBSCOhost Research Databases<br>Search Screen - Advanced Search<br>Database - CINAHL | 415 |

## **Table S3.** search strategy Embase

| | Search 22-03-2020 (after re-run) | |
|---|---|---|
| **Search** | **Query** | **Results** |
| #28 | ('virtual reality'/exp OR 'virtual reality' OR 'virtual reality exposure therapy' OR 'virtual reality simulator') AND ('pain'/exp OR pain OR 'pain management'/exp OR 'analgesia'/exp OR analgesia) AND ('burn'/exp OR burn OR 'burn patient' OR 'burn patient'/exp OR 'electric burn'/exp OR 'electric burn' OR 'chemical burn' OR 'chemical burn'/exp) AND ('preschool child' OR 'preschool child'/exp OR 'adolescent'/exp OR adolescent OR 'children'/exp OR children) | 48 |
| #27 | preschool child' OR 'preschool child'/exp OR 'adolescent'/exp OR adolescent OR 'children'/exp OR children | 4311987 |
| #26 | children | 1923545 |
| #25 | children'/exp | 2855524 |
| #24 | adolescent | 1745012 |
| #23 | adolescent'/exp | 1645129 |
| #22 | preschool child'/exp | 617266 |
| #21 | preschool child' | 617536 |
| #20 | burn'/exp OR burn OR 'burn patient' OR 'burn patient'/exp OR 'electric burn'/exp OR 'electric burn' OR 'chemical burn' OR 'chemical burn'/exp | 106550 |
| #19 | chemical burn'/exp ' | 4861 |
| #18 | chemical burn'/exp ' | 5102 |
| #17 | electric burn' | 2490 |
| #16 | electric burn'/exp | 2454 |
| #15 | burn patient'/exp | 4888 |
| #14 | burn patient' | 5609 |

| | | |
|---|---|---|
| #13 | burn | 101521 |
| #12 | burn'/exp | 78174 |
| #11 | pain'/exp OR pain OR 'pain management'/exp OR 'analgesia'/exp OR analgesia | 1723216 |
| #10 | analgesia | 216944 |
| #9 | analgesia'/exp | 167124 |
| #8 | pain management'/exp | 167124 |
| #7 | pain | 1309657 |
| #6 | pain'/exp | 1321444 |
| #5 | virtual reality'/exp OR 'virtual reality' OR 'virtual reality exposure therapy' OR 'virtual reality simulator' | 22291 |
| #4 | virtual reality simulator' | 834 |
| #3 | virtual reality exposure therapy' | 714 |
| #2 | virtual reality | 22291 |
| #1 | virtual reality'/exp | 16681 |

**Table S4.** screening process

# Results

## Pubmed (40)

| N° | Author | Title |
|---|---|---|
| 1 | Al-Ghamdi N.A. | Virtual Reality Analgesia With Interactive Eye Tracking During Brief Thermal Pain Stimuli: A Randomized Controlled Trial (Crossover Design). |
| 2 | Wang Y.L. | Immersive virtual reality as analgesia for women during hysterosalpingography: study protocol for a randomized controlled trial. |
| 3 | Hornsby N. | Psychosocial Interventions Targeting Recovery in Child and Adolescent Burns: A Systematic Review. |
| 4 | Wu W.W. | [Design and application of mobile soothing screen for dressing change of children with burns of limb]. |
| 5 | Hoffman H.G. | Immersive Virtual Reality as an Adjunctive Non-opioid Analgesic for Pre-dominantly Latin American Children With Large Severe Burn Wounds During Burn Wound Cleaning in the Intensive Care Unit: A Pilot Study. |
| 6 | Eijlers R. | Systematic Review and Meta-analysis of Virtual Reality in Pediatrics: Effects on Pain and Anxiety. |
| 7 | Wojciechowski E. | Feasibility of designing, manufacturing and delivering 3D printed ankle-foot orthoses: a systematic review. |
| 8 | Wolbrink T.A. | The Top Ten Websites in Critical Care Medicine Education Today. |
| 9 | Khadra C. | Projector-based virtual reality dome environment for procedural pain and anxiety in young children with burn injuries: a pilot study. |
| 10 | Scapin S.Q. | Use of virtual reality for treating burned children: case reports. |
| 11 | Gomez J. | The Use of Virtual Reality Facilitates Dialectical Behavior Therapy® "Observing Sounds and Visuals" Mindfulness Skills Training Exercises for a Latino Patient with Severe Burns: A Case Study. |
| 12 | Pardesi O. | Pain Management in Pediatric Burn Patients: Review of Recent Literature and Future Directions. |
| 13 | Ghanouni P. | Magnetic resonance-guided focused ultrasound treatment of extra-abdominal desmoid tumors: a retrospective multicenter study. |

| | | |
|---|---|---|
| 14 | Brown N.J. | Cost-Effectiveness of a Nonpharmacological Intervention in Pediatric Burn Care. |
| 15 | Knudsen M. | Computed tomography-guided radiofrequency ablation is a safe and effective treatment of osteoid osteoma located outside the spine. |
| 16 | Glazer T.A. | Transoral robotic surgery for obstructive sleep apnea: perioperative management and postoperative complications. |
| 17 | Rowe H. | A cluster randomised controlled trial of a brief couple-focused psychoeducational intervention to prevent common postnatal mental disorders among women: study protocol. |
| 18 | Jeffs D. | Effect of virtual reality on adolescent pain during burn wound care. |
| 19 | Liu L. | [Effectiveness and safety assessments of thoracoscopic thoracic tuberculosis clearance and internal fixation with bone grafting supported by digital technology]. |
| 20 | Liu G. | Ultrasound-guided intralesional diode laser treatment of congenital extratruncular venous malformations: mid-term results. |
| 21 | Brown N.J. | Play and heal: randomized controlled trial of Ditto™ intervention efficacy on improving re-epithelialization in pediatric burns. |
| 22 | Faber A.W. | Repeated use of immersive virtual reality therapy to control pain during wound dressing changes in pediatric and adult burn patients. |
| 23 | Brown N.J. | Efficacy of a children's procedural preparation and distraction device on healing in acute burn wound care procedures: study protocol for a randomized controlled trial. |
| 24 | Kipping B. | Virtual reality for acute pain reduction in adolescents undergoing burn wound care: a prospective randomized controlled trial. |
| 25 | Louw Q. | Measuring children's distress during burns dressing changes: literature search for measures appropriate for indigenous children in South Africa. |
| 26 | Schmitt Y.S. | A randomized, controlled trial of immersive virtual reality analgesia, during physical therapy for pediatric burns. |
| 27 | Miller M.K. | Multi-modal distraction. Using technology to combat pain in young children with burn injuries. |
| 28 | Mahrer N. | The use of virtual reality for pain control: a review. |
| 29 | Sharar S.R. | Applications of virtual reality for pain management in burn-injured patients. |
| 30 | Hoffman H.G. | Virtual reality pain control during burn wound debridement in the hydrotank. |
| 31 | Miller K. | The emergence of multi-modal distraction as a paediatric pain management tool. |
| 32 | Mott J. | The efficacy of an augmented virtual reality system to alleviate pain in children undergoing burns dressing changes: a randomised controlled trial. |
| 33 | Sharar S.R. | Factors influencing the efficacy of virtual reality distraction analgesia during postburn physical therapy: preliminary results from 3 ongoing studies. |
| 34 | Van Twillert B. | Computer-generated virtual reality to control pain and anxiety in pediatric and adult burn patients during wound dressing changes. |

| | | |
|---|---|---|
| 35 | Chan E.A. | Application of a virtual reality prototype for pain relief of pediatric burn in Taiwan. |
| 36 | Haik J. | The use of video capture virtual reality in burn rehabilitation: the possibilities. |
| 37 | Hoffman H.G. | Using FMRI to study the neural correlates of virtual reality analgesia. |
| 38 | Das D.A. | The efficacy of playing a virtual reality game in modulating pain for children with acute burn injuries: a randomized controlled trial [ISRCTN87413556]. |
| 39 | Hoffman H.G. | Effectiveness of virtual reality-based pain control with multiple treatments. |
| 40 | Hoffman H.G. | Use of virtual reality for adjunctive treatment of adult burn pain during physical therapy: a controlled study. |

# CINAHL (5)

| N° | Author | Title |
|---|---|---|
| 1 | Jeffs D. | Effect of virtual reality on adolescent pain during burn wound care. |
| 2 | Faber A.W. | Repeated use of immersive virtual reality therapy to control pain during wound dressing changes in pediatric and adult burn patients. |
| 3 | Schmitt Y.S. | A randomized, controlled trial of immersive virtual reality analgesia, during physical therapy for pediatric burns. |
| 4 | Mott J. | The efficacy of an augmented virtual reality system to alleviate pain in children undergoing burns dressing changes: a randomised controlled trial. |
| 5 | Hoffman H.G. | Virtual reality pain control during burn wound debridement in the hydrotank. |

## Embase (48)

| N° | Author | Title |
|---|---|---|
| 1 | Gates M. | Digital technology distraction for acute pain in children: A Meta-analysis. |
| 2 | Al-Ghamdi N.A | Virtual Reality Analgesia With Interactive Eye Tracking During Brief Thermal Pain Stimuli: A Randomized Controlled Trial (Crossover Design). |
| 3 | Hornsby N. | Psychosocial Interventions Targeting Recovery in Child and Adolescent Burns: A Systematic Review. |
| 4 | Eijlers R. | Systematic Review and Meta-analysis of Virtual Reality in Pediatrics: Effects on Pain and Anxiety. |
| 5 | Furness P.J. | Reducing Pain during Wound Dressings in Burn Care Using Virtual Reality: A Study of Perceived Impact and Usability with Patients and Nurses. |
| 6 | Hoffman H.G. | Immersive Virtual Reality as an Adjunctive Non-opioid Analgesic for Pre-dominantly Latin American Children With Large Severe Burn Wounds During Burn Wound Cleaning in the Intensive Care Unit: A Pilot Study. |
| 7 | Hansen J.K. | Sedation and analgesia during pediatric burn dressing change: A survey of American burn association centers. |
| 8 | Shaw M. | Proceedings #9: Immersive Virtual Reality Rehabilitation for Patients with Multiple Sclerosis. |
| 9 | Soltani M. | Virtual reality analgesia for burn joint flexibility: A randomized controlled trial. |
| 10 | Khadra C. | Projector-based virtual reality dome environment for procedural pain and anxiety in young children with burn injuries: A pilot study. |
| 11 | Arane K. | Virtual reality for pain and anxiety management in children |
| 12 | Scapin S.Q. | Use of virtual reality for treating burned children: case reports. |
| 13 | Pardesi O. | Pain management in pediatric burn patients: Review of recent literature and future directions. |
| 14 | Gonzalez M. | Water-friendly adjunctive virtual reality pain distraction for pediatric burn patients during wound debridement in the ICU tubroom. |
| 15 | Hoffman H.G. | Auditory interactivity task increases effectiveness of virtual reality pain distraction (with and without oculus rift VR goggles). |
| 16 | Hoffman H.G. | Virtual reality pain distraction of a severe pediatric burn patient during wound debridement in the ICU tank room: A case study. |
| 17 | Hoffman H.G. | Feasibility of articulated arm mounted Oculus Rift Virtual Reality goggles for adjunctive pain control during occupational therapy in pediatric burn patients. |
| 18 | Brown N.J. | Play and heal: Randomized controlled trial of Ditto™ intervention efficacy on improving re-epithelialization in pediatric burns. |
| 19 | Hoffman H.G. | Feasibility of articulated arm mounted Oculus Rift Virtual Reality goggles for adjunctive pain control during occupational therapy in pediatric burn patients. |

| | | |
|---|---|---|
| 20 | Jeffs D. | Effect of virtual reality on adolescent pain during burn wound care. |
| 21 | Faber A.W. | Repeated use of immersive virtual reality therapy to control pain during wound dressing changes in pediatric and adult burn patients. |
| 22 | Jeffs D. | Effect of virtual reality on adolescent pain during burn wound care. |
| 23 | Brown N.J. | Efficacy of a children's procedural preparation and distraction device on healing in acute burn wound care procedures: Study protocol for a randomized controlled trial. |
| 24 | Kipping B. | Virtual reality for acute pain reduction in adolescents undergoing burn wound care: A prospective. |
| 25 | Fusco H.N. | Development and feasibility of a novel gaming system for children with upper extremity burn. |
| 26 | VerLee S. | The utility of virtual reality in minimizing procedural distress with pediatric burn patients. |
| 27 | Miller K. | A novel technology approach to pain management in children with burns: A prospective randomized controlled trials. |
| 28 | Tropez-Arceneaux. L. | Pain and perceived exertion in pediatric burn patients during exercise with virtual reality. |
| 29 | Schmitt Y.S. | A randomized, controlled trial of immersive virtual reality analgesia, during physical therapy for pediatric burns. |
| 30 | Bayat A. | Analgesia and sedation for children undergoing burn wound care. |
| 31 | Sen S. | Review of burn injury research for the year 2009. |
| 32 | Miller K. | Multi-modal distraction. Using technology to combat pain in young children with burn injuries. |
| 33 | Miller K. | Multimodal distraction to relieve pain in children undergoing acute medical procedures. |
| 34 | Richard R. | Burn rehabilitation and research: Proceedings of a consensus summit. |
| 35 | Sharar S.R. | Applications of virtual reality for pain management in burn-injured patients. |
| 36 | Mott J. | The efficacy of an augmented virtual reality system to alleviate pain in children undergoing burns dressing changes: A randomised controlled trial. |
| 37 | Miller K. | The emergence of multi-modal distraction as a paediatric pain management tool. |
| 38 | Hoffman H.G. | Virtual reality pain control during burn wound debridement in the hydrotank. |
| 39 | Sharar S.R. | Factors Influencing the Efficacy of Virtual Reality Distraction Analgesia During Postburn Physical Therapy: Preliminary Results from 3 Ongoing Studies. |

| | | |
|---|---|---|
| 40 | Van Twillert B. | Computer-generated virtual reality to control pain and anxiety in pediatric and adult burn patients during wound dressing changes. |
| 41 | Chan E.A. | Application of a virtual reality prototype for pain relief of pediatric burn in Taiwan. |
| 42 | Haik J. | The use of video capture virtual reality in burn rehabilitation: The possibilities. |
| 43 | Hoffman H.G. | Using fMRI to study the neural correlates of virtual reality analgesia. |
| 44 | Das D.A. | The efficacy of playing a virtual reality game in modulating pain for children with acute burn injuries: a randomized controlled trial [ISRCTN87413556]. |
| 45 | Das D.A. | The efficacy of playing a virtual reality game in modulating pain for children with acute burn injuries: A randomized controlled trial [ISRCTN87413556]. |
| 46 | Hoffman H.G. | Virtual-reality therapy. |
| 47 | Hoffman H.G. | Effectiveness of virtual reality-based pain control with multiple treatments. |
| 48 | Hoffman H.G. | Virtual reality as an adjunctive pain control during burn wound care in adolescent patients. |

# Results without duplicates

## Primary screening: title and abstract (59)

| N° | Author | Title | Reviewer 1 | Reviewer 2 | Reviewer 3 | Reason of exclusion |
|---|---|---|---|---|---|---|
| 1 | Al-Ghamdi N.A. | Virtual Reality Analgesia With Interactive Eye Tracking During Brief Thermal Pain Stimuli: A Randomized Controlled Trial (Crossover Design). | 0 | 0 | 0 | Not the target intervention |
| 2 | Wang Y.L. | Immersive virtual reality as analgesia for women during hysterosalpingography: study protocol for a randomized controlled trial. | 0 | 0 | 0 | Not the target intervention |
| 3 | Hornsby N. | Psychosocial Interventions Targeting Recovery in Child and Adolescent Burns: A Systematic Review. | 0 | 0 | 0 | No primary research |
| 4 | Wu W.W. | [Design and application of mobile soothing screen for dressing change of children with burns of limb]. | 0 | 0 | 0 | Not the target language |
| 5 | Hoffman H.G. | Immersive Virtual Reality as an Adjunctive Non-opioid Analgesic for Pre-dominantly Latin American Children With Large Severe Burn Wounds During Burn Wound Cleaning in the Intensive Care Unit: A Pilot Study. | 1 | 1 | 1 | |
| 6 | Eijlers R. | Systematic Review and Meta-analysis of Virtual Reality in Pediatrics: Effects on Pain and Anxiety. | 0 | 0 | 0 | No primary research |
| 7 | Wojciechowski E. | Feasibility of designing, manufacturing and delivering 3D printed ankle-foot orthoses: a systematic review. | 0 | 0 | 0 | Not the target intervention |
| 8 | Wolbrink T.A. | The Top Ten Websites in Critical Care Medicine Education Today. | 0 | 0 | 0 | Not the target intervention |
| 9 | Khadra C. | Projector-based virtual reality dome environment for procedural pain and anxiety in young children with burn injuries: a pilot study. | 0 | 0 | 0 | Not the target intervention |
| 10 | Scapin S.Q. | Use of virtual reality for treating burned children: case reports. | 1 | 1 | 1 | |
| 11 | Gomez J. | The Use of Virtual Reality Facilitates Dialectical Behavior Therapy® "Observing Sounds and Visuals" Mindfulness Skills Training Exercises for a Latino Patient with Severe Burns: A Case Study. | 0 | 0 | 0 | Not the target intervention |
| 12 | Pardesi O. | Pain Management in Pediatric Burn Patients: Review of Recent Literature and Future Directions. | 0 | 0 | 0 | No primary research |
| 13 | Ghanouni P. | Magnetic resonance-guided focused ultrasound treatment of extra-abdominal desmoid tumors: a retrospective multicenter study. | 0 | 0 | 0 | Not the target intervention |
| 14 | Brown N.J. | Cost-Effectiveness of a Nonpharmacological Intervention in Pediatric Burn Care. | 0 | 0 | 0 | Not the target intervention |

| | | | | | | |
|---|---|---|---|---|---|---|
| 15 | Knudsen M. | Computed tomography-guided radiofrequency ablation is a safe and effective treatment of osteoid osteoma located outside the spine. | 0 | 0 | 0 | Not the target intervention |
| 16 | Glazer T.A. | Transoral robotic surgery for obstructive sleep apnea: perioperative management and postoperative complications. | 0 | 0 | 0 | Not the target intervention |
| 17 | Rowe H. | A cluster randomised controlled trial of a brief couple-focused psychoeducational intervention to prevent common postnatal mental disorders among women: study protocol. | 0 | 0 | 0 | Not the target intervention |
| 18 | Jeffs D. | Effect of virtual reality on adolescent pain during burn wound care. | 1 | 1 | 1 | |
| 19 | Liu L. | [Effectiveness and safety assessments of thoracoscopic thoracic tuberculosis clearance and internal fixation with bone grafting supported by digital technology]. | 0 | 0 | 0 | Not the target intervention |
| 20 | Liu G. | Ultrasound-guided intralesional diode laser treatment of congenital extratruncular venous malformations: mid-term results. | 0 | 0 | 0 | Not the target intervention |
| 21 | Brown N.J. | Play and heal: randomized controlled trial of Ditto™ intervention efficacy on improving re-epithelialization in pediatric burns. | 0 | 0 | 0 | Not the target intervention |
| 22 | Faber A.W. | Repeated use of immersive virtual reality therapy to control pain during wound dressing changes in pediatric and adult burn patients. | 1 | 1 | 1 | |
| 23 | Brown N.J. | Efficacy of a children's procedural preparation and distraction device on healing in acute burn wound care procedures: study protocol for a randomized controlled trial. | 0 | 0 | 0 | Not the target intervention |
| 24 | Kipping B. | Virtual reality for acute pain reduction in adolescents undergoing burn wound care: a prospective randomized controlled trial. | 1 | 1 | 1 | |
| 25 | Louw Q. | Measuring children's distress during burns dressing changes: literature search for measures appropriate for indigenous children in South Africa. | 0 | 0 | 0 | No primary research |
| 26 | Schmitt Y.S. | A randomized, controlled trial of immersive virtual reality analgesia, during physical therapy for pediatric burns. | 0 | 0 | 0 | Not the target intervention |
| 27 | Miller M.K. | Multi-modal distraction. Using technology to combat pain in young children with burn injuries. | 0 | 0 | 0 | Not the target intervention |
| 28 | Mahrer N. | The use of virtual reality for pain control: a review. | 0 | 0 | 0 | No primary research |
| 29 | Sharar S.R. | Applications of virtual reality for pain management in burn-injured patients. | 0 | 0 | 0 | No primary research |
| 30 | Hoffman H.G. | Virtual reality pain control during burn wound debridement in the hydrotank. | 0 | 0 | 0 | Not the target intervention |
| 31 | Miller K. | The emergence of multi-modal distraction as a paediatric pain management tool. | 0 | 0 | 0 | Not the target intervention |
| 32 | Mott J. | The efficacy of an augmented virtual reality system to alleviate pain in children undergoing burns dressing changes: a randomised controlled trial. | 1 | 1 | 1 | |
| 33 | Sharar S.R. | Factors influencing the efficacy of virtual reality distraction analgesia during postburn | 0 | 0 | 0 | Not the target |

| | | | | | | |
|---|---|---|---|---|---|---|
| | | physical therapy: preliminary results from 3 ongoing studies. | | | | intervention |
| 34 | Van Twillert B. | Computer-generated virtual reality to control pain and anxiety in pediatric and adult burn patients during wound dressing changes. | 1 | 1 | 1 | |
| 35 | Chan E.A. | Application of a virtual reality prototype for pain relief of pediatric burn in Taiwan. | 1 | 1 | 1 | |
| 36 | Haik J. | The use of video capture virtual reality in burn rehabilitation: the possibilities. | 0 | 0 | 0 | Not the target intervention |
| 37 | Hoffman H.G. | Using FMRI to study the neural correlates of virtual reality analgesia. | 0 | 0 | 0 | Not the target intervention |
| 38 | Das D.A. | The efficacy of playing a virtual reality game in modulating pain for children with acute burn injuries: a randomized controlled trial [ISRCTN87413556]. | 1 | 1 | 1 | |
| 39 | Hoffman H.G. | Effectiveness of virtual reality-based pain control with multiple treatments. | 1 | 1 | 1 | |
| 40 | Hoffman H.G. | Use of virtual reality for adjunctive treatment of adult burn pain during physical therapy: a controlled study. | 0 | 0 | 0 | Not the target population |
| 41 | Gates M. | Digital technology distraction for acute pain in children: A Meta-analysis. | 0 | 0 | 0 | No primary research |
| 42 | Furness P.J. | Reducing Pain during Wound Dressings in Burn Care Using Virtual Reality: A Study of Perceived Impact and Usability with Patients and Nurses. | 0 | 0 | 0 | Not the target outcome |
| 43 | Hansen J.K. | Sedation and analgesia during pediatric burn dressing change: A survey of American burn association centers. | 0 | 0 | 0 | Not the target intervention |
| 44 | Shaw M. | Proceedings #9: Immersive Virtual Reality Rehabilitation for Patients with Multiple Sclerosis. | 0 | 0 | 0 | Not the target population |
| 45 | Arane K. | Virtual reality for pain and anxiety management in children. | 0 | 0 | 0 | Not the target intervention |
| 46 | Gonzalez M. | Water-friendly adjunctive virtual reality pain distraction for pediatric burn patients during wound debridement in the ICU tubroom. | 0 | 0 | 0 | No full text available |
| 47 | Hoffman H.G. | Auditory interactivity task increases effectiveness of virtual reality pain distraction (with and without oculus rift VR goggles). | 0 | 0 | 0 | Not the target intervention |
| 48 | Hoffman H.G. | Virtual reality pain distraction of a severe pediatric burn patient during wound debridement in the ICU tank room: A case study. | 0 | 0 | 0 | Not the target intervention |
| 49 | Hoffman H.G. | Feasibility of articulated arm mounted Oculus Rift Virtual Reality goggles for adjunctive pain control during occupational therapy in pediatric burn patients. | 0 | 0 | 0 | Not the target intervention |
| 50 | Fusco H.N. | Development and feasibility of a novel gaming system for children with upper extremity burn. | 0 | 0 | 0 | No full text available |
| 51 | VerLee S. | The utility of virtual reality in minimizing procedural distress with pediatric burn patients. | 0 | 0 | 0 | Not the target intervention |
| 52 | Miller K. | A novel technology approach to pain management in children with burns: A prospective randomized controlled trials. | 0 | 0 | 0 | Not the target intervention |

| | | | | | | |
|---|---|---|---|---|---|---|
| 53 | Tropez-Arceneaux. L. | Pain and perceived exertion in pediatric burn patients during exercise with virtual reality. | 0 | 0 | 0 | No primary research |
| 54 | Bayat A. | Analgesia and sedation for children undergoing burn wound care. | 0 | 0 | 0 | No primary research |
| 55 | Sen S. | Review of burn injury research for the year 2009. | 0 | 0 | 0 | Not the target intervention |
| 56 | Miller K. | Multimodal distraction to relieve pain in children undergoing acute medical procedures. | 0 | 0 | 0 | Not the target intervention |
| 57 | Hoffman H.G. | Virtual reality as an adjunctive pain control during burn wound care in adolescent patients. | 1 | 1 | 1 | |
| 58 | Soltani M. | Virtual reality analgesia for burn joint flexibility: A randomized controlled trial. | 0 | 0 | 0 | Not the target intervention |
| 59 | Richard R. | Burn rehabilitation and research: Proceedings of a consensus summit. | 0 | 0 | 0 | Not the target intervention |

## Snowball methods: forward and backward (1)

| N° | Author | Title | Reason of exclusion |
|---|---|---|---|
| 1 | Hua Y. | The Effect of Virtual Reality Distraction on Pain Relief During Dressing Changes in Children with Chronic Wounds on Lower Limbs. | Not the target population |

## Secondary screening: full text (12)

| N° | Author | Title | Reviewer 1 | Reviewer 2 |
|---|---|---|---|---|
| 1 | Hoffman H.G. | Immersive Virtual Reality as an Adjunctive Non-opioid Analgesic for Pre-dominantly Latin American Children With Large Severe Burn Wounds During Burn Wound Cleaning in the Intensive Care Unit: A Pilot Study. | 1 | 1 |
| 2 | Scapin S.Q. | Use of virtual reality for treating burned children: case reports. | 1 | 1 |
| 3 | Jeffs D. | Effect of virtual reality on adolescent pain during burn wound care. | 1 | 1 |
| 4 | Faber A.W. | Repeated use of immersive virtual reality therapy to control pain during wound dressing changes in pediatric and adult burn patients. | Not the target population | Not the target population |
| 5 | Kipping B. | Virtual reality for acute pain reduction in adolescents undergoing burn wound care: a prospective randomized controlled trial. | 1 | 1 |
| 6 | Mott J. | The efficacy of an augmented virtual reality system to alleviate pain in children undergoing burns dressing changes: a randomised controlled trial. | Not the target intervention | Not the target intervention |
| 7 | Van Twillert B. | Computer-generated virtual reality to control pain and anxiety in pediatric and adult burn patients during wound dressing changes. | 1 | 1 |
| 8 | Chan E.A. | Application of a virtual reality prototype for pain relief of pediatric burn in Taiwan. | 1 | 1 |
| 9 | Das D.A. | The efficacy of playing a virtual reality game in modulating pain for children with acute burn injuries: a randomized controlled trial [ISRCTN87413556]. | 1 | 1 |
| 10 | Hoffman H.G. | Effectiveness of virtual reality-based pain control with multiple treatments. | Not the target intervention | Not the target intervention |
| 11 | Hoffman H.G. | Virtual reality as an adjunctive pain control during burn wound care in adolescent patients. | 1 | 1 |
| 12 | Hua Y. | The Effect of Virtual Reality Distraction on Pain Relief During Dressing Changes in Children with Chronic Wounds on Lower Limbs. | Not the target population | Not the target population |

# Rerun

## Pubmed (+1)

| N° | Author | Title | Reviewer 1 | Reviewer 2 |
|---|---|---|---|---|
| 1 | Phelan I. | A Mixed-Methods Investigation Into the Acceptability, Usability, and Perceived Effectiveness of Active and Passive Virtual Reality Scenarios in Managing Pain Under Experimental Conditions. | Not the target intervention | Not the target intervention |

## CINAHL (+1)

| N° | Author | Title | Reviewer 1 | Reviewer 2 |
|---|---|---|---|---|
| 1 | Schmitt Y.S. | A randomized, controlled trial of immersive virtual reality analgesia, during physical therapy for pediatric burns. | Duplicate | Duplicate |

## Embase (+0)

# Included articles

| N° | Author | Year | Title |
|---|---|---|---|
| 1 | Van Twillert B. | 2007 | Computer-generated virtual reality to control pain and anxiety in pediatric and adult burn patients during wound dressing changes. |
| 2 | Chan E.A. | 2007 | Application of a virtual reality prototype for pain relief of pediatric burn in Taiwan. |
| 3 | Das D.A. | 2005 | The efficacy of playing a virtual reality game in modulating pain for children with acute burn injuries: a randomized controlled trial [ISRCTN87413556]. |
| 4 | Hoffman H.G. | 2000 | Virtual reality as an adjunctive pain control during burn wound care in adolescent patients. |
| 5 | Hoffman H.G | 2019 | Immersive Virtual Reality as an Adjunctive Non-opioid Analgesic for Pre-dominantly Latin American Children With Large Severe Burn Wounds During Burn Wound Cleaning in the Intensive Care Unit: A Pilot Study. |
| 6 | Scapin S.Q. | 2017 | Use of virtual reality for treating burned children: case reports. |
| 7 | Jeffs D. | 2014 | Effect of virtual reality on adolescent pain during burn wound care. |
| 8 | Kipping B. | 2012 | Virtual reality for acute pain reduction in adolescents undergoing burn wound care: a prospective randomized controlled trial. |
